# *CTNNA3* is a tumor suppressor in hepatocellular carcinomas and is inhibited by miR-425

**DOI:** 10.18632/oncotarget.6978

**Published:** 2016-01-22

**Authors:** Bing He, Ting Li, Lei Guan, Fang-E Liu, Xue-Mei Chen, Jing Zhao, Song Lin, Zhi-Zhen Liu, Hu-Qin Zhang

**Affiliations:** ^1^ The Key Laboratory of Biomedical Information Engineering of Ministry of Education, School of Life Science and Technology, Xi'an Jiaotong University, Xi'an 710049, P.R. China; ^2^ The Center of Basic Medicine Teaching Experiments, School of Basic Medicine, Fourth Military Medicine University (FMMU), Xi'an 710032, P.R. China

**Keywords:** CTNNA3, miR-425, HCC, proliferation, invasion

## Abstract

Hepatocellular carcinoma (HCC) is a common and leading cause of death worldwide. Here, we identified that a cell-cell adhesion gene, *CTNNA3*, is a tumor suppressor in HCC. *CTNNA3* inhibited the proliferation, migration and invasion of HCC cell lines. In these cells, *CTNNA3* inhibited Akt signal, and in turn decreased the proliferating cell nuclear antigen (PCNA) and the matrix metallopeptidase MMP-9, and increased the cell cycle inhibitor p21^Cip1/Waf1^. Meanwhile, *CTNNA3* is inhibited by miR-425 in HCC. The miR-425 directly bound to the 3′UTR of *C*TNNA3 and inhibited its expression. The tumor suppressor function of *CTNNA3* and the oncogenic function of miR-425 were further confirmed in HCC cell xenograft in nude mice. The miR-425/*CTNNA3* axis may provide insights into the mechanisms underlying HCC, and contribute to potential therapeutic strategy of HCC.

## INTRODUCTION

Human hepatocellular carcinoma (HCC) is the second leading cause of cancer-related death, causing an estimated over half a million deaths annually [1, 2]. Metastasis is the leading cause of most cancer-related deaths [3], especially in HCC [4]. Adhesion-junction proteins, which are intercellular junctions abundant in normal epithelia and reduced in cancers [5], were found to contribute to carcinogenesis and metastasis of many types of cancer [6]. Adhesion-junctions are formed by transmembrane E-cadherin, β-catenin, and α-catenin [5]. α-catenin integrates adhesion-junctions with the actin cytoskeleton and could promote intercellular adhesion [5]. α-catenin is a putative tumor suppressor in myeloid leukemia [7], glioblastoma [8], skin cancer [9, 10] and breast cancer [11, 12]. Among the three α-catenin coding genes, *CTNNA1* participates the carcinogenesis of multiple types of cancers [12, 13], and mutates in HCC [14]. *CTNNA2* mutates in gastric cancer [15] and laryngeal carcinomas [16]. *CTNNA3* is a tumor suppressor and frequently mutates in laryngeal carcinomas [16] and low-expressed in urothelial carcinoma of the bladder [17]. The role of *CTNNA2* and *CTNNA3* in HCC haven't been found yet.

miRNAs are small, non-coding RNAs that negatively regulate the expression of target genes by mRNA degradation or translational repression [18]. miRNAs function as important regulators in cancer microenvironment [19, 20]. Many miRNAs, like miR-3127 [21], miR-494 [22], miR-42509 [23], participate the carcinogenesis of HCC by inhibiting their target genes. Therefore, miRNAs are also included in our study.

In this study, we analyzed the expression of the three α-catenin coding genes in HCC using microarray data of HCC samples and normal liver controls with bioinformatics methods and identified that *CTNNA3* was down-regulated in HCC. CCK8 and Transwell assays revealed that *CTNNA3* inhibited proliferation, migration and invasion of HCC cells. The silence of *CTNNA3* resulted in increased proliferating cell nuclear antigen (PCNA), decreased cell cycle inhibitor p21^Cip1/Waf1^ and Akt signal activation, as well as the increased matrix metallopeptidase MMP-9. miR-425 inhibited *CTNNA3* in HCC. miR-425 directly bound to the 3′untranslated region of *CTNNA3* and inhibited *CTNNA3* to promote the proliferation, migration and invasion of HCC cells.

## RESULTS

### *CTNNA3* was down-regulated in HCC

The comparison of gene expression between HCC and normal healthy controls indicated that *CTNNA3* was down-regulated (*P*-value < 0.05 and LgFC < −1) in 78 of 81 human HCC samples. The other two α-catenin coding genes, *CTNNA1* and *CTNNA2*, were not deregulated in the analyzed HCC dataset. Therefore, *CTNNA3* was selected for further investigation.

### *CTNNA3* inhibited HCC cell proliferation

**Figure 1:**
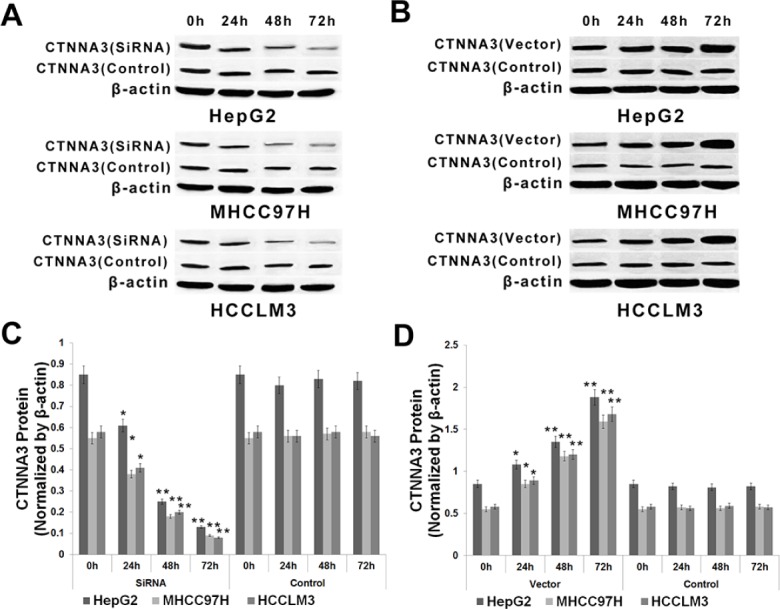
Expression of *CTNNA3* in HCC cells transfected with *CTNNA3* expression vector, siRNA or inactive controls (**A, C**) Protein of CTNNA3 decreased with time after transfection with *CTNNA3* siRNA in HepG2, MHCC97H and HCCLM3 cells. (**B, D**) Protein of CTNNA3 increased with time after transfection with *CTNNA3* overexpression vector in HepG2, MHCC97H and HCCLM3 cells; *p < 0.05, **p < 0.01, and ***p < 0.001.

**Figure 2:**
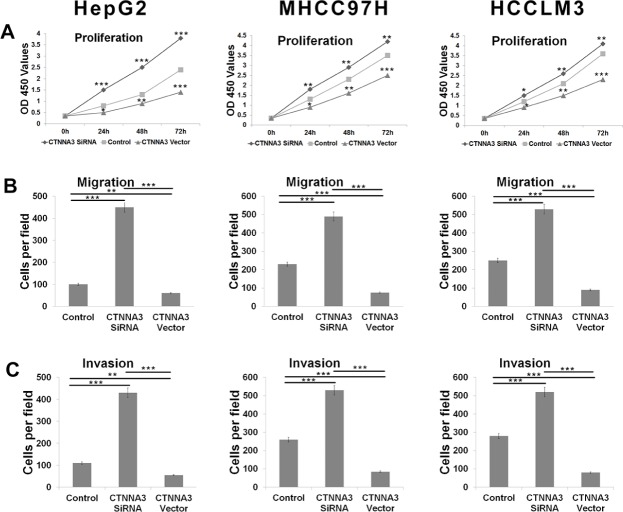
*CTNNA3* regulates HCC cell proliferation, migration and invasion (**A**) Growth of HCC cells was shown after transfection with *CTNNA3* siRNA or overexpression vector or inactive control. The growth index as assessed at 0, 24, 48 and 72 h. **(B)** Transwell analysis of HCC cells migration after treatment with *CTNNA3* siRNA or overexpression vector or inactive control. **(C)** Transwell analysis of HCC cells invasion after treatment with *CTNNA3* siRNA or overexpression vector or inactive control; *p < 0.05, **p < 0.01, and ***p < 0.001.

We then explored the potential impact of *CTNNA3* on HCC cell proliferation in HepG2, MHCC97H and HCCLM3 cell lines. HepG2, MHCC97H and HCCLM3 cells were transfected with *CTNNA3* overexpression vector or siRNA or inactive controls (Figure 1). CCK8 assay indicated that the cell proliferations were enhanced in all of the *CTNNA3*-siRNA-transfected HCC cell lines compared with inactive-control-transfected HCC cell lines (Figure 2A). Conversely, *CTNNA3* overexpression vector inhibited the cell proliferations of the HepG2, MHCC97H and HCCLM3 cells (Figure 2A).

### *CTNNA3* inhibited HCC cell cycle progression

**Figure 3:**
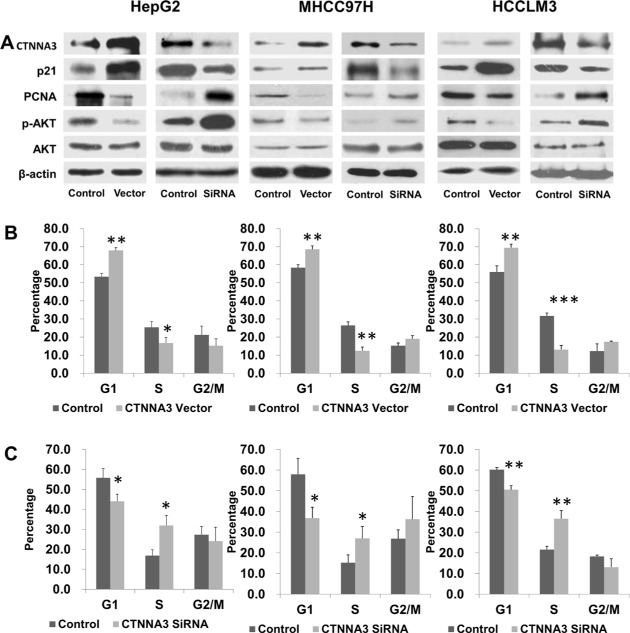
*CTNNA3* regulates HCC cell cycle progression (**A**) Changed expression of *CTNNA3* significant changed the levels of phosphorylated Akt, p21Cip1/Waf1 and PCNA **(B)** Cell cycle analysis of *CTNNA3* overexpression cells and control cells. **(C)** Cell cycle analysis of *CTNNA3* knockdown cells and control cells. *p < 0.05, **p < 0.01, and ***p < 0.001.

As *CTNNA3* inhibtied HCC cell proliferation, cell cycle analysis was performed to examine how *CTNNA3* affectes the cell cycle. Flow cytometric analysis showed that the percentage of *CTNNA3* overexpression cells at G1 phase increased comparing to control cells. This phenomenon was associated with a concomitant decrease of cells at the S phases of the cell cycle (Figure 3B). Moreover, the percentage of *CTNNA3* knockdown cells at G1 phase decreased comparing to control cells. And it was associated with a concomitant increase of cells at the S phases of the cell cycle (Figure 3C).

In order to investigate the mechanisms underlying the above changes in cell cycle progression, several cell cycle-related proteins were compared between *CTNNA3* overexpression cells, *CTNNA3* knockdown cells and control cells using western blot. Expression changes of *CTNNA3* did not cause significant deregulation of Cyclin A1, Cyclin A2, Cyclin D1, Cyclin D3 or Cyclin E2 (data not shown), but resulted in significant changes of the cell cycle inhibitor p21^Cip1/Waf1^ and the proliferation marker PCNA (Figure 3A). *CTNNA3* knockdown cells showed decreased protein levels of p21^Cip1/Waf1^ and increased protein levels of PCNA (Figure 3A). Conversely, *CTNNA3* overexpression cells showed increased protein levels of p21^Cip1/Waf1^ and decreased protein levels of PCNA (Figure 3A). These results supported the inhibition effect of *CTNNA3* on HCC cell cycle progression.

Since Akt signaling can lead to down-regulation of the CDK inhibitor p21^Cip1/Waf1^ [24], we examined the effect of *CTNNA3* on inhibiting Akt signaling by measuring the protein level of phosphorylated and unphosphorylated Akt. Western blot analysis showed that phosphorylated Akt was decreased in *CTNNA3* overexpression cells (Figure 3A), revealing that *CTNNA3* resulted in inhibited Akt signaling, which in turn leads to up-regulation of p21^Cip1/Waf1^ and concomitant down-regulation of PCNA.

### *CTNNA3* inhibited HCC cell migration and invasion

We explored the potential impact of *CTNNA3* on HCC cell migration and invasion in HepG2, MHCC97H and HCCLM3 cell lines. Cells were transfected with *CTNNA3* overexpression vector or siRNA or inactive controls. Transwell assay indicated that the cell migration and invasion were promoted in all of the *CTNNA3*-siRNA-transfected HCC cell lines compared with inactive-control-transfected HCC cell lines (Figure 2B and 2C). Conversely, *CTNNA3* overexpression vector could inhibit the migration and invasion of the HepG2, MHCC97H and HCCLM3 cells (Figure 2B and 2C).

**Figure 4:**
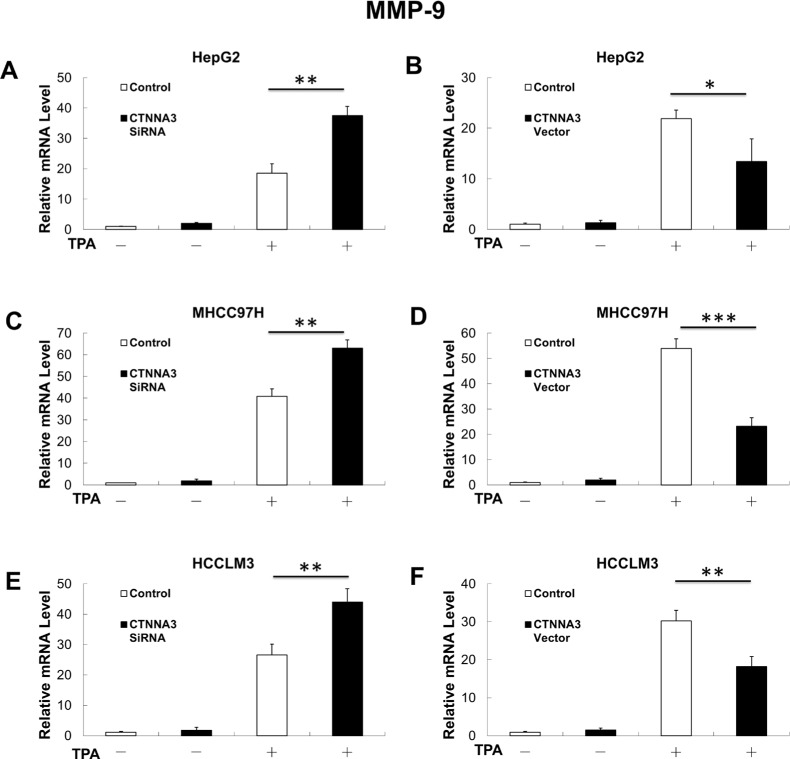
MMP-9 mRNA expression is negatively related with in *CTNNA3* HCC cells (**A**) TPA-induced MMP-9 mRNA expression was increased in *CTNNA3* knockdown HepG2 cells. **(B)** TPA-induced MMP-9 mRNA expression was decreased in *CTNNA3* overexpression HepG2 cells. **(C)** TPA-induced MMP-9 mRNA expression was increased in *CTNNA3* knockdown MHCC97H cells; **(D)** TPA-induced MMP-9 mRNA expression was decreased in *CTNNA3* overexpression MHCC97H cells. **(E)** TPA-induced MMP-9 mRNA expression was increased in *CTNNA3* knockdown HCCLM3 cells. **(F)** TPA-induced MMP-9 mRNA expression was decreased in *CTNNA3* overexpression HCCLM3 cells. *p < 0.05, **p < 0.01, and ***p < 0.001.

It has been reported that Akt promotes cancer cell invasion by increasing matrix metallopeptidase (MMP-9) [25]. This leads to the hypothesis that MMP-9 may also participate the molecular mechanisms underlying *CTNNA3* related inhibition of cell migration and invasion. Therefore, we investigated expression of MMP-9 in *CTNNA3* knockdown cells, *CTNNA3* overexpression cells and control cells with and without 12-O-tetradecanoylphorbol- 13-Acetate (TPA) treatment, which is an activator of protein kinase C that can promote cancer cell invasion via MMP-9 induction [26]. The results indicated that *CTNNA3* inhibited *MMP-9* in TPA-treated cells (Figure 4). These results indicated that *MMP-9* may be part of the molecular mechanisms that underlying the cell invasion inhibition by *CTNNA3* in HCC.

### MiR-425 targeted and inhibited *CTNNA3* in HCC cells

**Figure 5:**
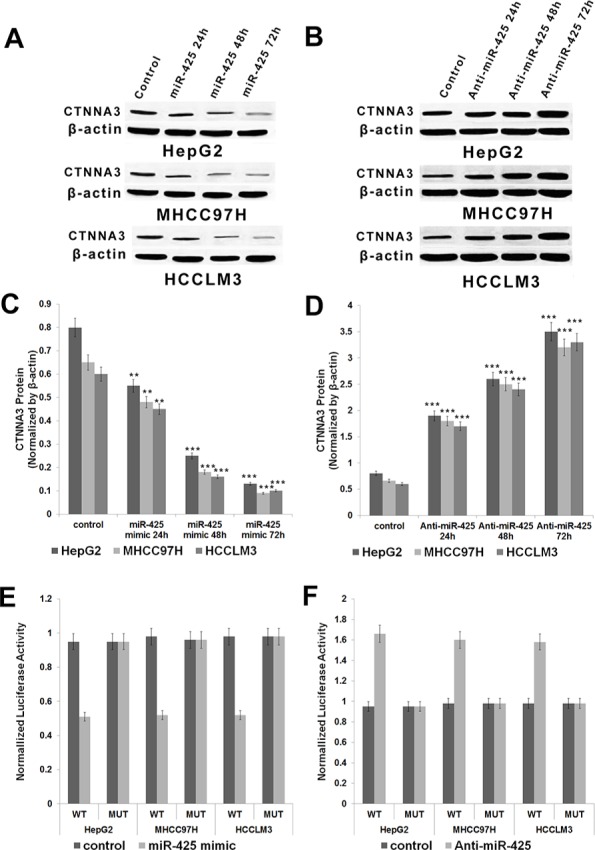
miR-425 targets and negatively regulates *CTNNA3* in HCC cells (**A**. **C**) Protein of CTNNA3 decreased with time after transfection with miR-425 mimics in HepG2, MHCC97H and HCCLM3 cells. (**B**, **D**) Protein of CTNNA3 increased with time after transfection with anti-miR-425 in HepG2, MHCC97H and HCCLM3 cells; **(E)** The analysis of the relative luciferase activities of *CTNNA3*-WT, *CTNNA3*-MUT in HepG2, MHCC97H and HCCLM3 cells after transfection with miR-425 mimics. **(F)** The analysis of the relative luciferase activities of *CTNNA3*-WT, *CTNNA3*-MUT in HepG2, MHCC97H and HCCLM3 cells after transfection with anti-miR-425; *p < 0.05, **p < 0.01, and ***p < 0.001.

As shown above, the down-regulation of *CTNNA3* may contribute to the carcinogenesis of HCC. Here come the question: How *CTNNA3* is down-regulated in human HCC? miRNAs are inhibitors of gene expression [18]. It lead to the hypothesis that *CTNNA3* might be inhibited by miRNA. Therefore, bioinformatics analysis was performed to predict the potential regulation interactions between miRNAs and *CTNNA3*. As predicted by miRanda [27] and TargetScan [28], 23 miRNAs have high complementarity to the 3′UTR of *CTNNA3*. Among them, only miR-425 was found to be up-regulated (*P*-value < 0.05 & LgFC > 0.5) in the 13 HCC samples. Therefore, miR-425 was selected for further validation using wet experiments. The results indicated that miR-425 reduced the protein levels of *CTNNA3* in HCC cells (Figure 5A and 5C), while miR-425 inhibitors increased the protein levels of *CTNNA3* in HCC cells (Figure 5B and 5D). Then the effect of miR-425 on the translation of *CTNNA3* mRNA into protein was assessed by a luciferase reporter assay (Figure 5E and 5F). miR-425 reduced the luciferase activity of the reporter gene with the wild type *CTNNA3* 3′UTR construct, but not with the mutant construct (Figure 5E). Meanwhile the miR-425 inhibitor enhanced the luciferase activity of the reporter gene with the wild type *CTNNA3* 3′UTR construct, but not with the mutant construct (Figure 5F). These evidences indicated that miR-425 inhibited *CTNNA3* by directly targeting its 3′UTR region.

### miR-425 promoted HCC cell proliferation, migration and invasion by inhibiting *CTNNA3*

**Figure 6:**
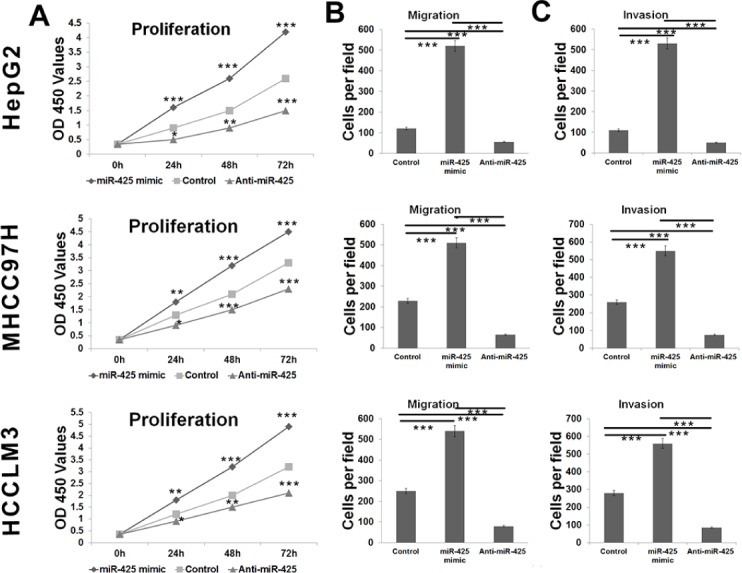
miR-425 promotes HCC cell proliferation, migration and invasion (**A**) Growth of HCC cells was shown after transfection with miR-425 mimics or inhibitor or inactive control. The growth index as assessed at 0, 24, 48 and 72 h. **(B)** Transwell analysis of HCC cells migration after treatment with miR-425 mimics, inhibitors or inactive control. **(C)** Transwell analysis of HCC cells invasion after treatment with miR-425 mimics, inhibitors or inactive control. *p < 0.05, **p < 0.01, and ***p < 0.001.

**Figure 7:**
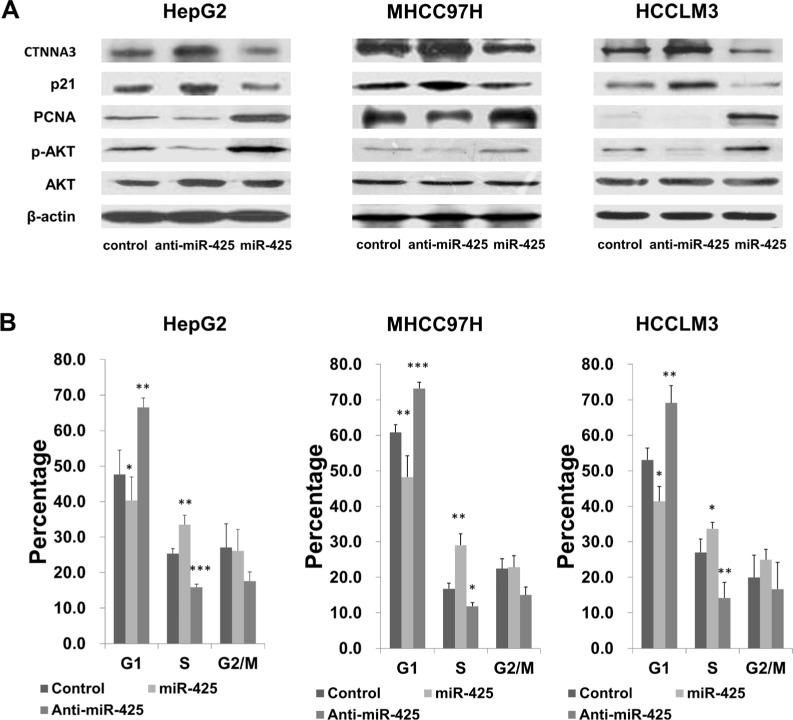
miR-425 regulates HCC cell cycle progression by inhibiting *CTNNA3* (**A**) miR-425 significant changed the levels of CTNNA3, phosphorylated Akt, p21Cip1/Waf1 and PCNA. **(B)** Cell cycle analysis of HCC cells transfected with miR-425 mimics or inhibitor or inactive control. *p < 0.05, **p < 0.01, and ***p < 0.001.

We explored the potential impacts of miR-425 on cell proliferation, migration and invasion in the HepG2, MHCC97H and HCCLM3 cell lines. HCC cells were transfected with miR-425 mimics or inhibitors or inactive control cel-mir-67, respectively. The results of CCK-8 proliferation assay revealed that the cell proliferation was promoted in all of the miR-425-mimics-transfected cell lines comparing to the inactive control cel-mir-67-transfected cell lines (Figure 6A). Meanwhile, miR-425 inhibitor decreased the proliferation of all the three HCC cell lines (Figure 6A). Flow cytometric analysis showed that miR-425 mimics promoted the transformation from G1 phase to S phases of the cell cycle (Figure 7B). At molecular level, the miR-425 mimics inhibited *CTNNA3*, and in turn resulted in increased PCNA, decreased p21^Cip1/Waf1^ and activated Akt (Figure 7A). These results revealed that the miR-425 promoted cell proliferation by inhibiting *CTNNA3*.

**Figure 8:**
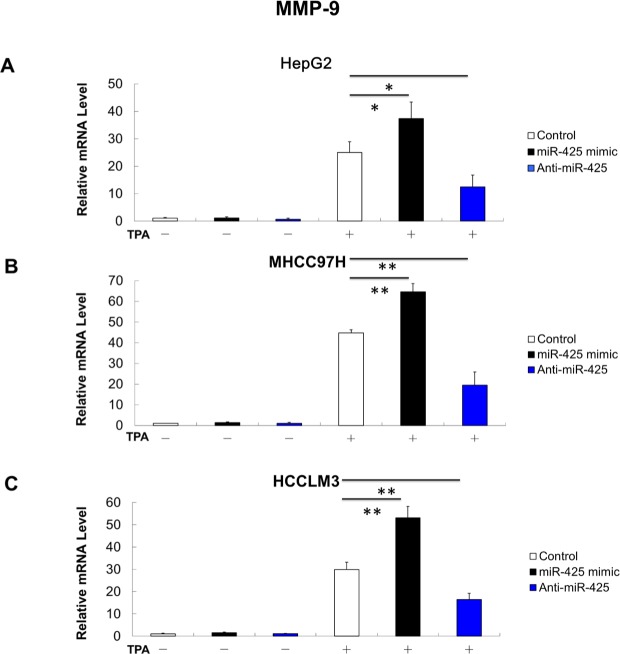
MMP-9 mRNA expression is positively related with miR-425 in HCC cells (**A**) MMP-9 mRNA expression in HepG2 cells with or without TPA treatment after transfection of miR-425 mimics or inhibitor or inactive control. **(B)** MMP-9 mRNA expression in MHCC97H cells with or without TPA treatment after transfection of miR-425 mimics or inhibitor or inactive control. **(C)** MMP-9 mRNA expression in HCCLM3 cells with or without TPA treatment after transfection of miR-425 mimics or inhibitor or inactive control. *p < 0.05, **p < 0.01, and ***p < 0.001.

Migration and invasion assay indicated that miR-425 mimics promoted the migration and invasion of HCC cells, whereas miR-425 inhibitors reduced migration and invasion of HCC cells (Figure 6B and 6C). At molecular level, the miR-425 mimics increased the mRNA levels of *MMP-9* in TPA-treated cells, while miR-425 inhibitors decreased the mRNA levels of *MMP-9* (Figure 8).

### *CTNNA3* suppresses the tumor growth of the HCC cell xenograft in nude mice

**Figure 9:**
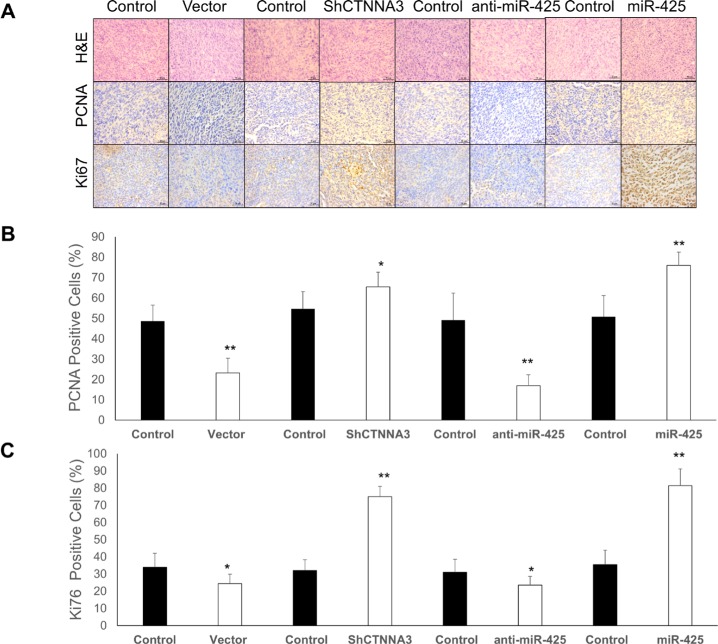
CTNNA3 suppresses the tumor growth of the HCC cell xenograft in nude mice (**A**) Representative image of tumors in nude mice after the injection of MHCC97H cells stably expressing *CTNNA3*, Sh*CTNNA3*, miR-425, anti-miR-425 and their controls (*n =* 8, 4 is shown). **(B)** Quantification of tumor weights of xenograft tumors in mice. **(C)** Growth curves of xenograft tumors in mice. *p < 0.05, **p < 0.01, and ***p < 0.001.

**Figure 10:**
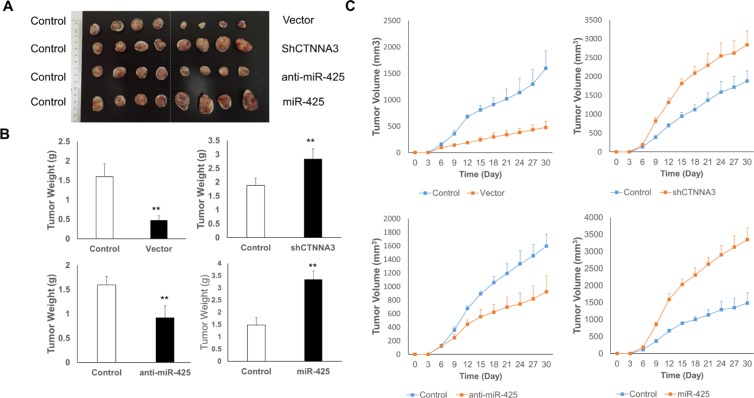
IHC staining for Ki67 and PCNA in the xenograft tumors in nude mice (**A**) Representative images of tumor samples that were stained with hematoxylin and eosin (H & E), Ki67, PCNA by IHC. **(B)** The percentages of PCNA-positive cells. **(C)** The percentages of Ki67-positive cells. *p < 0.05, **p < 0.01, and ***p < 0.001.

To explore the relationship between miR-425/*CTNNA3* axis and tumorigenesis *in vivo*, the xenograft model of HCC cells in nude mice was adopted. We used lentiviral vectors for the stable expression of *CTNNA3, CTNNA3* siRNA (Sh *CTNNA3*), miR-425 and miR-425 inhibitor (anti-miR-425) in MHCC97H cells. MHCC97H cells that stably expressed *CTNNA3*, Sh*CTNNA3*, miR-425, anti-miR-425, or their controls were injected subcutaneously into each flank of nude mice. The tumor weights and volumes were monitored. Then the growth curves of the tumors were plotted accordingly. We found that *CTNNA3* overexpression decreased tumor growth and miR-425 promoted tumor growth *in vivo* (Figure 9A–9C). We further performed IHC staining for Ki67 and PCNA in the tumors. Compared with the negative control, *CTNNA3* overexpression suppressed proliferative activity, as indicated by the percentage of cells positive for Ki67 and PCNA staining. In contrast, miR-425 overexpression increased the percentages of Ki67- and PCNA-positive cells (Figure 10). These *in vivo* observations confirmed the key role of miR-425/*CTNNA3* axis in the control of HCC cell growth and may serve as potential therapeutic targets for HCC treatment.

## DISCUSSION

HCC is the second leading cause of cancer-related death, causing an estimated over half a million deaths annually [1, 2]. Theα-catenin coding genes, *CTNNA1, CTNNA2* and *CTNNA3*, participate carcinogenesis of multiple cancers, such as laryngeal carcinomas [16], oropharyngeal squamous cell carcinomas [29] and breast cancer [12]. In this study, we analyzed gene microarray data of HCC samples and normal liver controls using bioinformatics methods with a special focus on the three α-catenin coding genes. The results indicated that *CTNNA3* was down-regulated in HCC (LgFC < −1).

In order to explore the function of *CTNNA3* in HCC, we silenced the expression of *CTNNA3* in HCC cell lines using siRNAs (Figure 1A and 1C). An important finding of this study is that *CTNNA3* inhibited the proliferation marker PCNA, implying that *CTNNA3* is an important proliferation inhibitor of HCC. It was supported by the fact that silence of *CTNNA3* in HCC cells results in increased cell proliferation (Figure 2A). Moreover, *CTNNA3* inhibited the cell cycle in G1-S and increased p21^Cip1/Waf1^ (Figure 3). Several studies showed that p21^Cip1/Waf1^ inhibits HCC cell proliferation, and p21^Cip1/Waf1^ could be inhibited by the Akt signaling pathway [24]. These findings are consistent with our results (Figure 3). These observations indicated that *CTNNA3* suppresses HCC cell proliferation at least in part through inhibiting the Akt signaling and in turn, increasing p21^Cip1/Waf1^.

Invasion and metastasis are characteristic features of HCC and major poor prognostic factor in HCC patients. Degradation of the extracellular matrix and the basement membrane is necessary for invasion and metastasis. *CTNNA3* encodes an adhesion-junction protein. It explains why *CTNNA3* inhibits the migration and invasion of the HCC cells. Moreover, it's interesting to find that *CTNNA3* inhibits HCC invasion not only by itself. MMP-9, which plays an important role in the proteolytic destruction of the extracellular matrix and basement membranes, was found to be inhibited by *CTNNA3* (Figure 4). MMP-9 would be increased by Akt signaling pathways [30]. In this study, we showed that *CTNNA3* inhibits the Akt signaling in HCC cells. Therefore, *CTNNA3* may inhibit cell invasiveness by inhibiting MMP-9 via Akt signaling pathway.

Then we explored the potential mechanism of impaired *CTNNA3* expression in HCC. The miRNAs, which inhibit gene expression and function as important regulators in cancer microenvironment [20, 31], were selected as the research focus. Bioinformatics analysis indicated the potential regulatory interaction between miR-425 and *CTNNA3*. Luciferase assay and western blot confirmed that miR-425 directly targets the 3′UTR region of *CTNNA3* and inhibit its expression (Figure 5). The inhibition of *CTNNA3* by miR-425 in turn resulted in increased PCNA, decreased p21^Cip1/Waf1^ and Akt activation (Figure 7A), as well as the increased MMP-9 (Figure 8). CCK-8 and transwell assay showed that miR-425 promoted cell proliferation, migration and invasion of HCC cell lines (Figure 6). Moreover, miR-425 promotes the tumor growth of the HCC cell xenograft in nude mice, while *CTNNA3* suppresses the growth. These results revealed that miR-425 promotes cell proliferation, migration and invasion by inhibiting *CTNNA3* in HCC.

In general, our study highlights the crucial role of *CTNNA3* in human HCC by inhibiting cell proliferation and invasion. And *CTNNA3* is inhibited by miR-425. The miR-425/*CTNNA3* axis provide insights into the mechanisms underlying human HCC, and may be involved in potential therapeutics of HCC.

## MATERIALS AND METHODS

### Gene and miRNA profile data collection

Gene and miRNA microarray data of HCC and normal control samples were collected from GEO database (http://www.ncbi.nlm.nih.gov/gds, GSE62232, GSE67882, and GSE40744). After quality control with limma package on R platform, 81 patients with HCC and 10 healthy controls were selected for gene expression analysis, 13 HCC patients and 15 healthy controls were selected for miRNA expression analysis.

### Identification of differentially expressed genes and miRNAs

The differentially expressed genes and miRNAs in patients with HCC were identified with Limma package on R platform using download gene and miRNA microarray data as mentioned above. The cutline for differentially expressed gene and miRNA is *P* value < 0.05 (*T*-test).

### miRNA target genes prediction

Human miRNA target genes prediction were performed using miRNA sequences downloaded from the Rfam website (http://www.sanger.ac.uk/Software/Rfam) on January 2015. A uniform system for microRNA annotation was used to retrieve 3′UTR sequences data for human genes [32] RepeatMasker [33] was used to mask repetitive elements in these sequences. The miRanda [27] and TargetScan [28] methods were used to predict the target genes of miRNAs. The predicted target genes that supported by both of the methods were selected for further analysis.

### HCC cell lines and cell culture

The human HCC cell lines HepG2, MHCC97H and HCCLM3 was obtained from the Chinese Center for Type Cultures Collections (CCTCC, Wuhan, China). The HepG2, MHCC97H and HCCLM3 cell lines were cultured in RPMI 1640 media (Life Technologies, Beijing, China) and supplemented with 10% fetal bovine serum (FBS) (Life Technologies, Beijing, China). All the Cells were maintained at 37°C in a humidified atmosphere with 5% CO2.

### Cell transfection

Full-length *CTNNA3* coding sequences were amplified and cloned into a pcDNA^™^ 3.1 expression vector (Life Technologies, Beijing, China), following manufacturer's protocol. HepG2, MHCC97H and HCCLM3 cell lines were seeded in 24-well plates at 3 × 10^5^ cells/wells and incubated overnight. Transfection of the *CTNNA3* pcDNA^™^ 3.1 vectors, inactive control mock pcDNA^™^ 3.1 vectors, *CTNNA3* siRNAs, inactive control scrambled siRNAs, miR-425 miRNA mimics, anti-miR-425, inactive control cel-mir-67 (Life Technologies, Beijing, China), or pMIR-Report vectors were taken using Lipofectamine 2000 transfection reagent (Life Technologies, Beijing, China) with 1 μg/ml DNA plasmid or 300 nmol of miRNA, respectively. Total proteins of HepG2, MHCC97H and HCCLM3 cells were isolated at 24 h, 48 h and 72 h after transfection.

### Cell migration and invasion assay

Cell invasion and migration were detected using a transwell chamber assay (Corning, Beijing, China) with or without Matrigel (Life Technologies, Beijing, China). For the determination of HepG2, MHCC97H and HCCLM3 cell invasion, transwell chamber was placed into a 24-well plate, then coated with 30 μl Matrigel, and incubated at 37°C for 40 minutes. In transwell assay with or without Matrigel, HepG2, MHCC97H and HCCLM3 cells were trypsinized and seeded in chambers at the density of 6 × 10^4^ cells/well at 48 h after the transfection. These cells were cultured in RPMI 1640 medium with 2% serum. And 600 μl of 10% FBS-1640 was added to the lower chamber. After 24 h, migrated HepG2, MHCC97H and HCCLM3 cells were fixed in the 100% methanol for 30 minutes. Those non-migrated HepG2, MHCC97H and HCCLM3 cells were removed by cotton swabs. After that cells on the bottom surface of the membrane were fixed and stained with the 0.1% crystal violet for 30 minutes. Images of HepG2, MHCC97H and HCCLM3 cells were taken using a phase-contrast microscope.

### Cell proliferation assay

Cell proliferation was detected using a Cell Counting Kit-8 assay (Dojindo, Kumamoto, Japan). HepG2, MHCC97H and HCCLM3 cells were plated in 24-well plates at 3 × 10^5^ cells/well. Then HCC cells were incubated at 37^°^C in 10% CCK-8 diluted with normal culture medium for color conversion. Proliferation rate was measured at 24 h, 48 h and 72 h after transfection.

### Cell cycle analysis

6 × 10^5^ cells were synchronized by serum starvation for 24 h. Then the cells were induced to re-enter the cell cycle by changing with 10% fetal bovine serum for 24 h. floating and adherent cells were harvested and fixed using 75% ethanol overnight at 4°C. After that, cells were incubated with RNase A for 30 min at 37°C. Then cells were stained with propidium iodide. Cell cycle was determined by flow cytometry.

### Luciferase assay

HepG2, MHCC97H and HCCLM3 cells were seeded in 24-well plates at 3 × 10^5^ cells/well and then incubated for 24 hours. Then the HepG2, MHCC97H and HCCLM3 cells were co-transfected with 0.6 μg of pGL3-CTNNA1–3′UTR or pGL3-CTNNA1–3′UTR Mut plasmid, or 0.06 ng of the phRL-SV40 control vector (Promega, Beijing, China), and 100 nM miR-425 mimics or miR-425 inhibitors or control RNA using Lipofectamine 2000 (Invitrogen, Beijing, China). The renilla and firefly luciferase activities were measured by a dual luciferase assay (Promega, Beijing, China) at 24 h after transfections.

### Western blot

Isolated proteins from HepG2, MHCC97H and HCCLM3 cells at 24 h, 48 h and 72 h after transfections were separated by 12% SDS-PAGE gel and transferred onto nitrocellulose membranes (Bio-Rad, Beijing, China). Membranes were blocked with 5% non-fat milk and incubated with anti-CTNNA3 antibody (Abcam, Beijing, China) or anti-β-actin antibody (Abcam, Beijing, China). After extensive washes, the secondary antibody (Abcam, Beijing, China) was added to the system. Finally, Immunoreactive protein bands were determined using the Enhanced Chemiluminescence (ECL) system.

### Real-time reverse transcription quantitative PCR

Total RNA was extracted with Trizol reagent (Invitrogen, Beijing, China) according to the instructions. The cDNA was synthesized from total RNA with MMLV reverse transcriptase (Invitrogen, Beijing, China) and random hexamers. Real-time PCRs were performed using ABI 7300 Sequence Detection System (Life, Beijing, China). Relative quantification was determined by normalization to the amount of GAPDH. Primers used for real-time PCR were designed by Primer Express 3.0 and synthesized in Invitrogen.

### *In vivo* tumorigenicity assay

Tumor cells (5 × 10^6^) were injected subcutaneously into each flank of 4-week-old male Balb/c athymic nude mice (*n* = 8 per group). Mice were monitored every three day and euthanized one month later. Then tumors were dissected and weighed. The tumor volume was calculated as V (volume, mm3) = 0.5 × L (length, mm) × W^2^ (width, mm^2^).

### Immunohistochemistry (IHC)

IHC was performed on tissue microarray chips (Outdo, Shanghai, China). Single serial sections were made from xenograft tumor samples. The slides were probed with the following primary antibodies: mouse anti–human Ki67, and mouse anti–human PCNA (Abcam, Beijing, China). Then the slides were incubated with HRP-conjugated goat anti–mouse secondary antibodies. The proteins were visualized *in situ* with DAB chromogenic substrate.

### Statistical analysis

Experiments were repeated at least three times to achieve confident results. Statistical analysis was performed using R. All the data were presented as means ± S.D. Differences between groups were determined with Student's *t*-test. Statistical analysis was considered to be significant when *P* value < 0.05.
